# Rudolf Virchow: Integrating Medicine and Social Reform for Public Health

**DOI:** 10.7759/cureus.68161

**Published:** 2024-08-29

**Authors:** Pratik P Tawde, Sonali G Choudhari, Zahiruddin Quazi Syed, Abhay Gaidhane

**Affiliations:** 1 Department of Preventive and Community Medicine, Jawaharlal Nehru Medical College, Datta Meghe Institute of Higher Education and Research, Wardha, IND

**Keywords:** public health, anthropology, social medicine, cellular pathology, historical vignette

## Abstract

Rudolf Virchow, also known as Rudolf Carl Virchow, was a physician, pathologist, medical scientist, anthropologist, politician, social reformer, and role model. However, he is best known as the founder of the field of cellular pathology. He is known as "the father of modern pathology" and the founder of social medicine. He was born on October 13, 1821, in Prussia (now Swidwin, Poland) and died on September 5, 1902, in Berlin, Germany. He stressed that most diseases of mankind can be understood in terms of the dysfunction of cells. His study subjects were cell theory, disease, embolus, and thrombosis. He actively promoted social reforms and helped establish anthropology as a contemporary scientific field. He was also awarded and honored by the Copley Medal in 1892 for his notable work in “Cellular Pathology as Based Upon Physiological and Pathological Histology” and “Handbuch der Speziellen Pathologie und Therapie.” Virchow said, “Medicine is a social science, and politics is nothing more than medicine on a grand scale.” He believed that politics and social structures could have a significant positive or negative impact on public health, that medicine and public health practices used politically might change society, and that politicians and doctors had a moral duty to improve society. Knowing about Virchow helps us appreciate his ideas that laid the groundwork for many medical and scientific practices, the historical development of medical science, and the ongoing need to address social health factors. Virchow's contributions are still relevant in today's medical and public health fields. His work on cellular pathology forms the basis for many aspects of contemporary medicine, such as cancer, infectious diseases, and genetic disorders. His focus on social determinants of health remains a core principle in public health. Today, issues such as poverty, education, housing, and nutrition are acknowledged as factors affecting health outcomes. Virchow's beliefs in ethical responsibility, social transformation, and justice have affected medical ethics and the role of health professionals in society. This article highlights Rudolf Virchow's enormous contribution to pathology, medicine, and public health.

## Introduction and background

**Figure 1:**
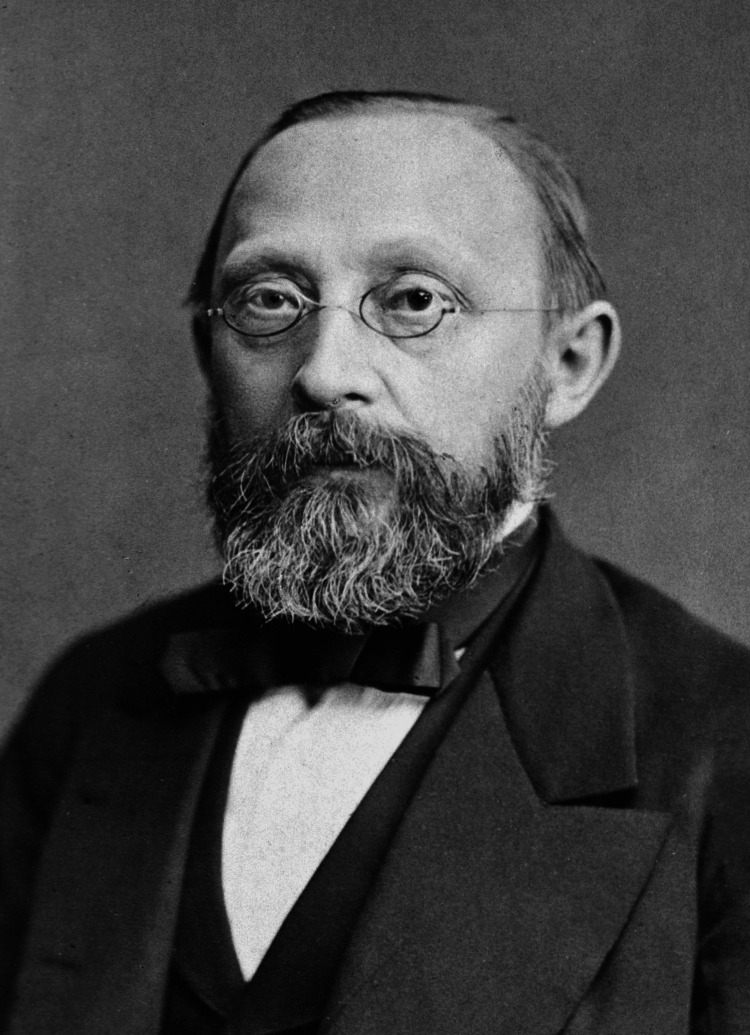
Picture of Rudolf Ludwig Carl Virchow Source: [7] Copyright/license: The noncommercial reuse of this content is free and open to all

The primary aim of this article is to highlight Rudolf Virchow's (Figure 1) immense contributions to pathology, medicine, and public health. Rudolf Ludwig Carl Virchow, a German physician, is often called "the father of modern pathology" due to his research that significantly advanced medical science by challenging the concept of humorism. He also founded Social Medicine and Veterinary Pathology and was called the "Pope of Medicine" by his colleagues. He coined the term zoonosis and quoted that "between animal and human medicine," there are no dividing lines, nor should there be [1,2]. This quote from Virchow can be related to the origins of One Health. Many people have found inspiration in Virchow's dual profession. He is frequently acknowledged for being among the first to argue for the complex genesis of epidemics and the social causes of sickness [3,4]. He was a well-known scientist who openly advocated for social medicine, public health reform, and political engagement, serving as a powerful symbol, hero, and inspiration [5,6].

## Review

Early life and career

Rudolf Ludwig Karl Virchow was born on October 13, 1821, in Schivelbein, Pomerania, Poland, and passed away on September 5, 1902. His parents were modest, and his father worked as a farmer and a retail merchant. During his youth, Virchow was educated at a parochial school in Schivelbein, where he also received extra teaching from the nearby clergymen. He then enrolled at the gymnasium in Koslin and finished his studies in 1838 at the age of 17 [8].

He studied medicine at Friedrich Wilhelm University in Berlin, Germany, in 1839 after receiving a scholarship. After defending his thesis titled "De rheumate praesertim corneae" and graduating in 1844, he further assisted Robert Froriep, a pathologist interested in microscopy. Because of Rudolf's academic achievements, he was granted the chance to deliver a speech at Berlin's Military Medical Academy's 50th-year event in 1845, where he discussed the "accuracy of medicine from a mechanical perspective" [9].

In 1845, at 23 years old, he authored a document on thrombosis and hemostasis that introduced the factors contributing to blood clot formation, known as Virchow's triad. By the end of that year, he also explained leukemia. The journal Archive for Pathological Anatomy and Physiology and Clinical Medicine was coedited by Virchow and Reinhardt until Reinhardt passed away in 1852 and then solely edited by Virchow until his passing in 1902 [10].

Virchow keened interest and developed ideas in social medicine and politics after getting a picture of patients being affected by the typhus epidemic due to poverty as one of the causes when he was offered employment to study the epidemic by the Prussian government during 1947-1848. Rudolf Virchow's study on the typhus outbreak of 1848 is considered a classic that has been largely overlooked. His examination of the outbreak focused on the economic, social, and cultural elements contributing to its cause and accurately pinpointed the conflicting social pressures that hindered a straightforward resolution [11]. He also helped establish Medical Reform, a weekly magazine advocating social medicine to address political injustices. Later, he was suspended from his official position in 1849, thus discontinuing the publication [12].

In November 1848, he was appointed to hold Germany’s first chair of pathological anatomy at the University of Würzburg. During his seven-year tenure, he worked scientifically on a six-volume Handbook on Special Pathology and Therapeutics, cellular theory, and thrombosis. For the next 20 years, he was the director and newly elected chair of the Institute of Pathology at Charité [13,14].

Virchow's contribution

Virchow contributed in various fields during his career. His main focused subjects were pathology, medicine, and public health.

Cell Biology

Cell theory is Virchow's most famous contribution to science, which he further developed on the contribution of Theodor Schwann. Virchow first accepted Robert Remak's study of the origin of cells. Later, there was a drift between Remak and Virchow when they published Remak's work after not initially accepting it. Virchow further extended and stated that a living cell gives rise to another living sir after Francesco Redi introduced Omne vivum ex ovo [15].

Cancer

In 1847, Virchow classified the ailment as a blood disorder and named it leukämie. In 1857, he was the first to describe a particular kind of cancer known as a chordoma from the clivus [16].

Virchow differentiated the origin of cancer cells from normal cells and suggested that dormant cell activation gives rise to cancer. He observed that white blood cells, which caused inflammation, were related to some cancers. His theory of chronic irritation was ignored and proven wrong, and it was later described as metastasis [17].

The Kaiser's Case

Kaiser Frederick III was suggested for removal of the entire larynx as he was suffering from cancer of the larynx. Virchow was against this, as the whole larynx was never successfully removed. In 1887, Virchow performed many biopsy tests and labeled the tissue noncancerous. After Kaiser's death, postmortem reports confirmed that the tissue was epidermal carcinoma. Virchow's name was not added to the report of Kaiser Frederick III by Bergmann and was criticized and accused of misdiagnosis and malpractice [18].

Thromboembolism

Virchow is also credited with developing the terms embolism and thrombosis and explaining the process of pulmonary thromboembolism in 1859 after working on autopsies. Later, he started to work on his hypothesis, which was that pulmonary thrombi are carried from the veins in the leg through the bloodstream, which can transport such a clot. He planned trials based on that theory, which were carried out repeatedly to gather data with an extremely precise approach. This was also the origin of Virchow's triad [19].

Pathology

Virchow was very fond of the microscope, and he thus encouraged German medical students to use the microscope and think microscopically. He was also the founder of cellular and comparative pathology. He also published Die Cellularpathologie in ihrer Begründung auf physiologische und pathologische Gewebelehre. The origins of One Health can be related to Virchow, who coined the term zoonoses, where he established a link between humans and animals [20].

Parasitology

Virchow declared that worms were also capable of causing human helminthiasis after examining the life cycle of *Trichinella spiralis*, which he discovered in the muscle tissue of deceased humans and dogs. He further demonstrated that if heated before, infected meat was infective to dogs and humans. Berlin was the first city to adopt meat inspection based on this [21].

Autopsy

Virchow conducted many autopsies and developed a systematic method for autopsy. He also published a book on his method. He performed several important postmortems, including one in 1845, where he discovered high levels of white blood cells in a 50-year-old woman, which he later identified as leukemia; another in 1856 on a baby, where he was the first to document congenital pulmonary lymphangiectasia; and another in 1857, where he described vertebral disc rupture [22,23].

During an autopsy conducted on May 8, 1884, Virchow identified the clinical syndrome known as ochronosis in a 67-year-old man. This syndrome was characterized by the build-up of homogentisic acid in connective tissues and was detectable by discoloration visible under a microscope. Something of such kind was observed for the first time [24].

Forensic Medicine

In 1861, Virchow produced the first forensic report analyzing hair in a criminal case. He was the first to acknowledge that hair alone cannot serve as proof. He discovered that each individual's hair can differ, that each hair has unique characteristics, and that hairs from several individuals can remarkably resemble one another [25].

Darwinism

Virchow did not endorse Darwin's theory of evolution because it was yet to be proven and lacked empirical foundations. Therefore, teaching Darwin's theory of evolution in schools could negatively impact scientific studies. He expressed his views publicly in Munich in "The Freedom of Science in the Modern State" [26].

Antigerm Theory

Virchow believed that some cell activities were responsible for diseases rather than pathogens. Hence, he was not supportive of the germ theory of diseases. He also believed that political strategies should be used over medical strategies to fight an epidemic. He considered poverty one of the main social variables of disease [27].

Politics and social medicine

Virchow was not just a lab doctor but a fervent supporter of political and social change. According to his beliefs, diseases are caused by social inequality, which calls for political action, for which he promoted social reform to combat illness and poverty. Most of his techniques revolved around observing pathology and statistics, which he labeled social medicine [28].

His political beliefs are clear in his Report on the Typhus Epidemic in Upper Silesia, where he claims that radical action to advance the advancement of an entire population is achievable only through "full and unlimited democracy" and "education, freedom, and prosperity" is the only way to end the outbreak rather than medicating individual patients or making small changes to food, housing, or clothing laws. He made advancements in water and sewer systems. Thus, he was recognized as the father of social medicine and anthropology [12,29]. With the COVID-19 pandemic, tuberculosis, maternal and child mortality, primary health care, One Health, and other global health issues at hand, Virchow's theory emphasizing the importance of social and environmental factors for health is especially pertinent now due to their significant sociopolitical impact on prevention and control policies.

Relations With Bismarck

As a cofounder and member of the liberal party Deutsche Fortschrittspartei, Virchow was one of Bismarck's primary political opponents. His criticism of Bismarck's extravagant military spending infuriated Bismarck so much that he challenged Virchow to a duel in 1865. Virchow refused, seeing dueling as an antiquated means of resolving disputes [30]. Later, Virchow helped Bismarck in his efforts to lessen the Catholic Church's societal and political sway, which he termed Kulturkampf [31].

Personal life and achievements

Virchow was a perceptive and truthful scholar. Despite his busy work schedule, others generally liked him well, and he never seemed rushed. He was a dedicated educator who excelled at teaching. He had no time for sports or hobbies, although he did like music and the occasional beer. During his vacations, he loved to go hiking and would frequently record formal observations of different phenomena while out for walks. Although he supported vivisection, he had empathy for the animals used in experiments. His handwriting was bad, and his offices were constantly disorganized. He completed the majority of his papers in one sitting. Despite having a lot of self-confidence, he declined the honorific title "von Virchow" [10]. Virchow married Ferdinande Rosalie on August 24, 1850, in Berlin. They had three daughters and three sons named Karl, Hans, Adele, Ernst, Marie, and Hanna [32].

Death

On January 4, 1902, while Virchow was getting off the electric tramway, he leaped from a running carriage and broke his thigh bone. Despite his expectations of a full recovery, his femur fracture never healed and limited his range of motion. His condition steadily declined, and on September 5, 1902, in Berlin, he passed away from heart failure following an eight-month illness [33].

Honors, legacy, and work

Virchow was honored with many posts and awards in his lifetime, even after his death. He also worked on several studies during his career, of which few were published. His honors and legacy are mentioned in Table 1 in chronological order, and the studies he worked on are mentioned in Table 2 [8,34-37].

**Table 1:** Yearwise honors and legacy of Rudolf Virchow Source: [8,34-37] Compiled by the author Pratik P. Tawde

Year	Honors and legacy
1859	Chosen as a member of the Berlin Chamber of Representatives
1860	Elected as an official member of the Royal Scientific Board for Medical Affairs
1861	Elected in the Royal Swedish Academy of Sciences
1862	Chosen as an international member of the American Philosophical Society
	Elected to the Prussian House of Representatives.
1873	Elected in the Prussian Academy of Sciences.
1880	Chosen as a member of the Reichstag of the German Empire
1881	On the occasion of his 60th birthday, the Rudolf Virchow Foundation was established
1892	Was named the Head of Berlin University
1910	A limestone statue of Rudolf Virchow was erected at Karlplatz
1915	Langenbeck-Virchow-Haus was built in Berlin, jointly honoring Virchow and Bernhard von Langenbeck
2002	The Biomedical Research Centre, named Rudolf Virchow Centre, was established at the University of Würzburg

**Table 2:** Yearwise work done by Rudolf Virchow Source: [8,34-37] Compiled by the author Pratik P. Tawde

Year	Work done
1848	Information on the typhus epidemic prevailing in Upper Silesia
1859	Cellular pathology in its foundation on physiological and pathological tissue teaching
1854-1876	Handbook of special pathology and therapy
1862-1872	Lectures on pathology
1863-1867	The malignant tumors
1868	About typhus fever
1875	Some characteristics of lower human races on the skull
1876	Contributions to the physical anthropology of the Germans
1877	The freedom of science in the modern state
1879	Collected essays from the field of public medicine and the study of epidemics
1890	Against antisemitism

## Conclusions

Virchow had a natural curiosity and a commitment to intellectual honesty. He put in a lot of effort, being a dedicated teacher who cared deeply for his students. He liked music but had no free time for sports or hobbies. He tied the knot with Rose Mayer in 1850 and had six children. On his holidays, he liked to go hiking and frequently recorded formal observations of different phenomena while walking. He supported vivisection yet displayed empathy toward animals used in experiments. His workspaces were consistently messy, and he had bad handwriting. He completed the majority of his papers in a single sitting. He was very self-assured but declined the special title "von Virchow." Despite his issues with Bismarck, he was extremely patriotic. He was pragmatic in several ways, his pragmatism is evident in his approach to medicine, science, and public health. Virchow's pragmatism was characterized by a focus on practical, evidence-based solutions and a holistic understanding of the factors affecting health. Despite working as a pathologist without gloves or a mask, he remained in good health for almost his entire life. His career aligned with significant advancements in microscope quality and the application of dyes for tissue staining. Virchow emphasized the importance of doctors advocating for the poor and having a responsibility to society, particularly to the most disadvantaged members of society. Many people have found inspiration in Virchow's dual profession. He is frequently acknowledged as having been among the first to argue for the complex etiology of epidemics and the social roots of sickness. However, because he was a prominent scientist and an outspoken supporter of the social foundation of medicine, public health reform, and political involvement, he has also functioned as a potent icon, hero, and role model.
